# An education programme influencing health professionals to recommend exercise to their type 2 diabetes patients – understanding the processes: a case study from Oxfordshire, UK

**DOI:** 10.1186/s12913-017-2040-7

**Published:** 2017-02-11

**Authors:** Anne Matthews, Natasha Jones, Alastair Thomas, Perdy van den Berg, Charlie Foster

**Affiliations:** 1 Nuffield Department of Population Health, University of Oxford, Old Road Campus, Oxford, OX3 7LF UK; 2 Department of Sports and Exercise Medicine, Nuffield Orthopaedic Centre, Windmill Road, Oxford, OX3 7HE UK; 3 Generation Games, AgeUK Oxfordshire, West St Helen Street, Abingdon, OX14 5BT UK; 4 Oxfordshire Community Diabetes Service, Victoria House Surgery, 119 Buckingham Road, Bicester, OX26 3EU UK

**Keywords:** Medical education, Health professional, Exercise, Behaviour change, Type 2 diabetes

## Abstract

**Background:**

Increasing levels of physical activity decreases the risk of premature mortality associated with chronic diseases e.g., coronary heart disease, type 2 diabetes, stroke. Despite this, most adults in England do not meet physical activity guidelines. Physical activity advice and signposting offered to at-risk patients by primary care providers is recommended. However, exercise medicine education is sparse, leading to poor practitioner knowledge of the risk reduction evidence and strategies to implement effective patient behaviour change. The ‘Generation Games’ intervention seeks physical activity increase in the 50+ population of Oxfordshire. It offers a Health Professional Education Programme (HPEP) providing exercise medicine education, and promotion of Generation Games to which health professionals can signpost patients. There is a poor evidence base concerning how such education translates into patient exercise behaviour change.

**Methods:**

The research aimed to create more understanding of how an education programme can influence health professionals to recommend Generation Games to and increase exercise behaviour in type 2 diabetes patients. A case study method facilitated examination of the routines and cultures studied – the experience of Diabetes nurses was used as an example of best practice engagement with the HPEP. Observation, interviews and documentation were employed to triangulate data. Data analysis refined and developed themes within key theoretical frameworks.

**Results:**

Firstly, there is a lack of knowledge about physical activity risk reduction benefits and a belief that efforts to motivate patients to increase their physical activity are ineffective, thus creating barriers to engagement with the HPEP. Secondly, practice nurses tasked with delivering lifestyle advice to diabetes patients – themselves suffering a motivational interviewing skill deficit – find ingrained physical activity behaviours extremely challenging, and therefore highly value the HPEP for providing helpful tools. Thirdly, patients who hear of Generation Games from a health professional may have mismatched expectations of how their exercise behaviour can change.

**Conclusions:**

Exercise medicine education has the potential to improve patient care and services. Before initiatives like the HPEP can succeed, primary care practice requires a more supportive exercise medicine culture. Also necessary is adequate resourcing of patient-centred behaviour change advice, training, encouragement and monitoring services.

## Background

Physical inactivity worldwide is responsible for 6% of the incidence of coronary heart disease, 7% of type 2 diabetes, 10% of breast cancer, and 10% of colon cancer [1]. In England (2011), only 13% of men and 7% of women aged 65+ achieved the recommended level of physical activity (150 min of moderate activity per week and muscle-strengthening activities on at least 2 days per week) [2]. As physical activity levels decline with age, the risk of ill-health, decreased physical functioning, and greater number of falls increases. Four million hospital day beds in England each year are accounted for by falls and fractures in those aged 65+ [3]. Whilst the physical activity guidance for older adults is clear, the challenge remains to increase uptake of physical activity within this group.

Inactivity has a greater impact on adults who have conditions such as type 2 diabetes. Of all the risk factors for type 2 diabetes – age, genetics, weight and ethnicity – weight (being overweight or obese) is the most important, since obesity accounts for 80-85% of the risk of developing the condition [4]. The rise in the prevalence of type 2 diabetes has led campaigners to advocate that the principal combating strategy should be to tackle the rise in obesity, through healthy lifestyle advocacy including ‘promotion of physical activity’ [5]. Increased physical activity, independent of weight loss, reduces the incidence of Type 2 diabetes [6].

Guidance from the National Institute for Health and Clinical Excellence (NICE) indicates that ‘brief advice’ from health professionals ‘has a modest, but consistent, positive effect on physical activity levels’[7], a strategy receiving government backing in the light of rising National Health Service (NHS) healthcare costs [8]. However, the practice of offering physical activity advice is currently not part of normal primary healthcare routines [9]. Recent evidence cites a number of barriers to health professionals offering ‘brief advice’. Primarily these include a lack of time, poor knowledge and training in exercise medicine education and behaviour change techniques, previous lack of success with changing patient behaviour, and lack of incentives to promote physical activity; if health practitioners were active themselves, this was an ‘enabler’ to the offering of ‘brief advice’ [10]. In England, exercise medicine education is not routinely part of medical training, nor is there a comprehensive professional development programme in place to provide healthcare professionals with risk reduction knowledge or behaviour change training [11].

Further, NICE advises that Care Pathway structures e.g., cardio-vascular disease, type 2 diabetes and stroke, should integrate exercise provision [12]. Currently, pathway provision structures do not support patients’ physical activity – rehabilitation schemes are fragmented and incomplete, lacking the resources to address exercise barriers. Provision beyond rehabilitation shows pathways as ‘often suboptimal leading to poor long-term adherence’ [13].

The Generation Games (GG) intervention aims to offer such integration by delivering exercise medicine education to health professionals through the Health Professional Education Programme (HPEP), encouraging them to offer exercise advice to their patients and providing a mechanism of provision of exercise to which they can refer. Following recommendation of GG to patients, and then contact being made by the patient, signposting from GG to appropriate exercise opportunities occurs by phone or letter. Secondary care pathway providers, having engaged with the GG offer (perhaps through attending an HPEP session), also signpost their patients – often in one-to-one settings – to pathway-specific exercise provision promoted by GG. The evidence base about the processes involved in such interventions is sparse, particularly in respect of the physical activity behaviour change in older populations. Other complex interventions have highlighted that changes in behaviour must incorporate both knowledge and motivation to change [14]. It is also unclear which educational delivery mode factors facilitates the greatest positive changes in professional practice [15, 16], given that health professionals may operate within unsupportive healthcare systems and cultures.

### An overview of the health professional education programme

Generation Games developed an educational package for health professionals under the guidance of Sport and Exercise Medicine experts at the Nuffield Orthopaedic Centre, Oxford. In the first year a feedback questionnaire was used to assess learning and improve programme content and delivery.

The content of the programme is tailored specifically to the audience concerned, as there are differing levels of understanding, current activity and therefore need across the care pathways and variety of health professionals to whom it is delivered. The programme as delivered contains the following elements in principle:

1. The science and statistics behind why physical activity matters for health
2. Physical activity and condition-specific risk reduction e.g., diabetes, cardiovascular disease, falls
3. Generation Games promotion and offer
4. Physical activity behaviour change in patients – discussion of techniques and challenges

Sessions (lasting between 10 min and 1½ hours) are usually ‘one-off’ although a few practices have received more than one session. The mode of delivery is always interactive including a tailored PowerPoint presentation. Participants may be given Generation Games sign-up cards or Exercise at Home DVDs to take away. The education programme was supported by a comprehensive education and information resource on the Generation Games website for health professionals. At the time of the study over 1300 health professionals had received the programme, including 270 GPs and their practice nurses, and over 500 other health professionals reaching twenty one different care pathways.

## Methods

This study took place as one aspect of a wider evaluation of the GG programme, seeking to answer the following research question:

‘How can we understand the processes at work, from health professional engagement with the HPEP to increasing the physical activity of Generation Games participants.’

**Fig. 1:**
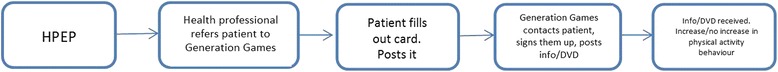
Flowchart showing pathway of behaviours necessary to ‘reach’ a Generation Games participant

A case study research design was adopted given its suitability for examining processes and its utilisation of thematic analysis to facilitate understanding of aspects of routine and culture [17]. Figure 1 summarises the assumptions behind the behavioural processes within the HPEP concept. A number of ‘interventions’ are necessary by a variety of ‘players’ before the participant is ‘reached’ by GG. Process evaluations – such as that outlined in Fig. 1 – have been highlighted as important in shedding light on the complexities of public health interventions, since they have the capacity to examine causal mechanisms and contextual factors. Participants may interact with interventions according to their circumstances, attitudes, beliefs, social norms and resources [18]. For this study, it was important to learn more about the experiences, attitudes and beliefs of those involved. For example, if a central tenet of the HPEP concept is to reach GG participants via health professionals – who currently do not routinely offer exercise advice to their patients – then it was important to learn more about the barriers which prevent them doing so.

Similarly, patients may well hear that they ‘should’ increase their physical activity, but it is known that levels of exercise declines with age. It is therefore important to learn more about how older people interpret and rationalise such messages in the context of their subsequent behaviour. Thus, the study examined:

1. education tutors’ experience of programme delivery, approaches to a variety of health professionals, and outcome expectations.
2. health professionals’ understanding and experience of behaviour change strategies, existing knowledge and receptivity to education about the importance of physical activity, and how the HPEP training had contributed to these professional aspects.
3. older adults with diabetes and current physical activity, any barriers faced, and their engagement, interpretation and response to the GG exercise message.

The HPEP is delivered to a wide range of health professionals and pathways. In order to gain a deeper understanding of the processes at work from delivery to outcome, one particular healthcare pathway – Diabetes – was selected as a focus for a number of reasons. Firstly, plentiful data sources would be optimised as there was already an established relationship between the Oxfordshire Community Diabetes Service (OCDS) and GG. Secondly, the OCDS had engaged with the HPEP on several prior occasions, thereby providing opportunities for considering ‘best practice’ features of the delivery mode of the programme. Thirdly, the GG database contained several participants with type 2 diabetes who potentially could be invited to participate in the case study.

### Data collection

Data were collected from multiple sources:

1. *Observation*
  Observation included the delivery of the HPEP to the OCDS (two 1½ hour education sessions to district and practice nurses), and the delivery of one 3-hour OCDS diabetes2gether structured group education session. Unstructured observational notes were taken covering relevant session areas - content, tutor style and delivery, and attendee responses. Efforts to counter observer bias were made by discussing the noted key points with tutors post-observation.

2. *Interviews*
  Interviews were undertaken with the HPEP programme tutor (one interview), the Generation Games programme manager (one interview), the OCDS Clinical Lead (one joint interview), a new OCDS diabetes nurse (one joint interview), four practice nurses (one interview with each), four diabetes patients who were also Generation Games participants but not patients of any health professionals in the case study (one interview with each)

3. *Documentation*
  Documentation consisted of course feedback, handouts, and Generation Games programme notes. All documents were obtained via Generation Games tutors and provided a useful background to how the programme was run and monitored. The course feedback formed part of the data analysis in establishing attendee opinions of course tutors.

### Data analysis

Analysis followed a case description model [19] which uses analytic techniques represented in matrices, flowcharts and diagrams to build up a descriptive framework. The data were examined for causal links between the education programme delivery and physical activity outcomes in diabetes patients. Such an approach facilitates describing the multiplicity of decisions and events which occur from education programme conception and delivery to behaviour outcomes of both health professionals and patients. Explanations about the case are systematically developed through repeated re-examination using the devised frameworks set out below [17].

### Theoretical framework

**Fig. 2:**
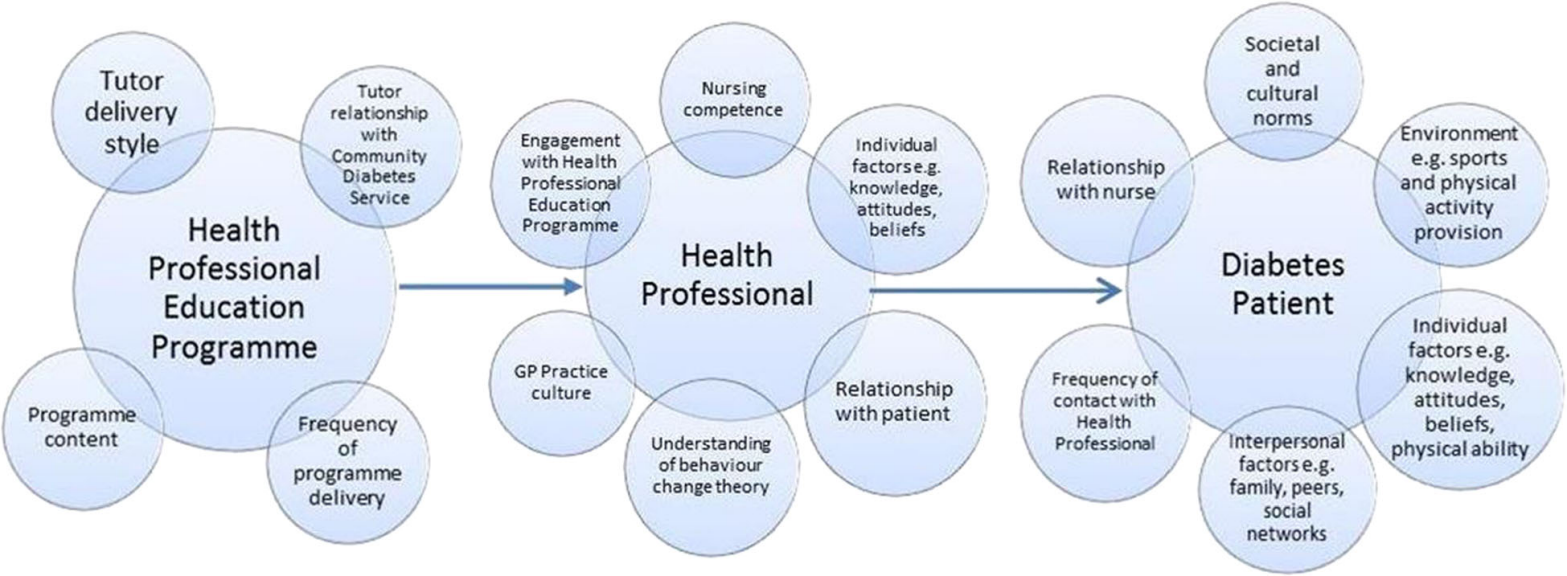
Flowchart showing Generation Games Health Professional Education Programme

The processes set out in Fig. 1 can be thought of as a ‘bounded system’, separated out for the purposes of creating limits to what is being studied [20]. The presence of a theoretical framework within the study, provides a way of strengthening the validity of the findings, by using theory to frame explanations of events, circumstances and behaviours [21, 22]. Figure 2 highlights the complex environment which surrounds the HPEP, and the multiplicity of factors which come into play surrounding the programme itself e.g., tutor style, relationship with client group, programme content and frequency. The flowchart highlights the interaction between with HPEP and practice nurses who attend sessions to update their diabetes training (organised by the Oxfordshire Community Diabetes Service).

**Fig. 3:**
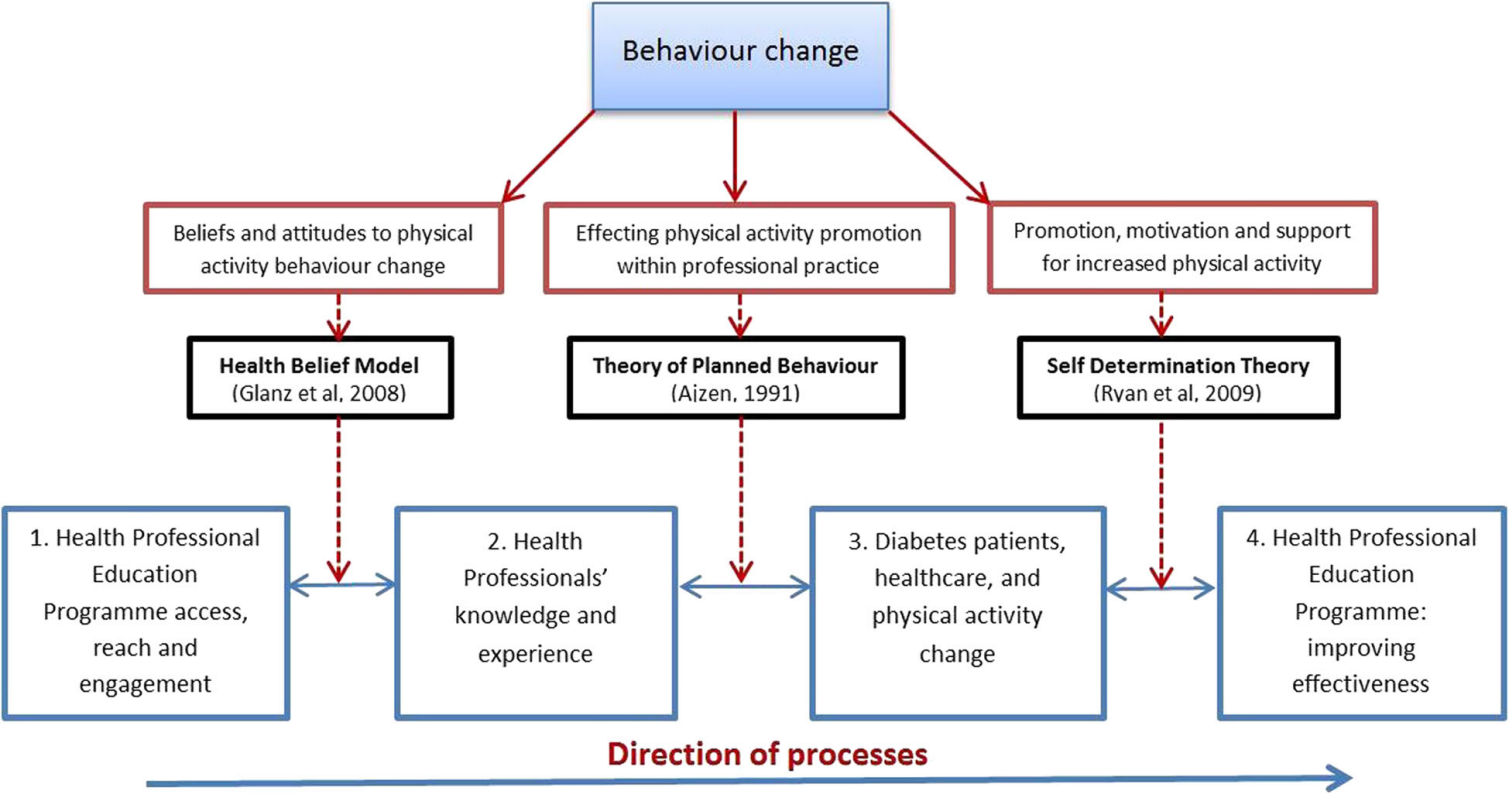
Flowchart showing four aspects central to the HPEP concept of patient ‘reach’, and three theoretical models used for data analysis

A theoretical framework was developed from Fig. 2 to show four domains of interest, predicated on the extent to which behaviour change can be effected at various stages of the process (see Fig. 3). Referenced are three well established theoretical models:

- *Health Belief model* – relating to health professionals beliefs about patient attitudes to exercise. This model was chosen as one of the most widely used for understanding health behaviour
- *Theory of Planned Behaviour model* – relating to health professional behaviour. This model was chosen to examine the relationship between beliefs and behaviour in healthcare settings
- *Self Determination Theory model* – relating to patient exercise behaviour. This model was chosen for its focus on the individual’s psychological needs and how they affect motivation

The case study illuminates both professional and patient behaviour change issues – both shown to be important in order to increase the likelihood of changes in physical activity, and the potential for Generation Games to be an effective agent.

## Results

### Engagement with the health professional education programme

#### Barriers to reach and access

About 60/65% of practices within Oxfordshire have had contact with the HPEP [Interview, HPEP Clinical Lead]. Gaining access to GPs was challenging, with personal contacts being the most fruitful route.

*‘I email the practice manager initially. If I get no reply I email again. And if they still don’t reply, I look down the list of GPs and see if there’s anyone I know, and if there is, I’ll email them again and copy in that doctor…But my knowledge of the doctors tends to be the thing that gets me in…’ [Interview, HPEP Clinical Lead]*

Part of the difficulty gaining access may reflect the neglect of physical activity within primary care [23]. The lack of an incentive to ‘reward’ health care professionals for attention to such a well-recognised risk factor for many chronic diseases is thought to contribute to this situation.

*‘There’s nothing in the GP contract to ask them to [attend sessions like HPEP]. This is an extra.’ [Interview, HPEP Clinical Lead]*

The ‘reach’ of the HPEP to the healthcare pathways has proved easier than to GP practices. It seems likely that here, the programme offer is seen as more relevant in content and usefulness in terms of behaviour change information and signposting for patients.

*‘They’re much more easy to engage. Well, for the rehab pathways, they already understand exercise. You don’t have to convince them about that. A lot of them have this whole ‘what am I going to do with the patients when they come off the end of the pathway.’ So they’re looking for an answer for that… they genuinely believe in the value of exercise rehabilitation.’ [Interview, HPEP Clinical Lead]*

### HPEP content and delivery

It is appreciated that little is known about the effect of different factors regarding the education of health professionals in respect of patient physical activity [24] – likely influences at the point of delivering the education are the quality, context and content, and the deliverer themselves [25]. For GPs, the ‘context’ is nearly always subject to the pressure of time, and therefore the HPEP presentation is focused on ‘case study’ patients for whom physical activity would be a beneficial recommendation – sometimes as little as 10 min is given to the HPEP deliverer within a GP practice setting. In terms of content, GPs commonly are resistant to the session at first.

*‘The first 5 min they look at you a bit blankly, “Why am I sitting here? Why am I not doing something else.”’ [Interview, HPEP Clinical Lead]*

Despite occasional reluctance to engage at the beginning, the session usually becomes interactive and discussion reveals to GPs the potential of exercise – as therapy or prevention – which they may not have properly considered before

*‘And they realise they actually don’t know a lot of the statistics behind this. So they don’t know the risk reduction statistics so they can’t pass that on to patients…GPs at that point tend to get more engaged. Then when you start talking about patients, and they start thinking, ‘I don’t know that’ and this is actually quite interesting, that’s when [the session] usually gets going.’ [Interview, HPEP Clinical Lead]*

HPEP education sessions tend to be lengthier for healthcare pathway professionals than for time-poor GPs. For them, the HPEP presentation content is always pathway-relevant. Sessions organised by the OCDS – updating practice nurses about diabetes treatment – last around 1½ hours and occur off-site. A continuing relationship between the OCDS and the HPEP has enabled development and adaptation of the programme to tailor what is delivered to the context of diabetes.

*‘One of the real benefits of the presentation to nurses, is that it actually puts into context the specific benefits to glucose regulation from activity, rather than just cardiovascular benefit. That bit’s been taken out of the presentation, and we’ve asked for it to be put back in.’ [Interview, OCDS Clinical Lead]*

The inclusive nature of the programme content and an enthusiastic presentation style appear to be important factors for success. Such an approach has inspired adaptation within the OCDS’s own professional approach.

*‘[The Clinical Lead] is a brilliant ambassador for the programme and the physio [HPEP exercise insructor] who is inspirational…They’re such good advocates. And actually, the feedback from the courses when the two of them have been delivering the sessions has been better than the others…[The Clinical Lead is] very good at involving different groups of people.*

*We’ve struggled for a long time and thinking ‘are there any tricks we can use around behavioural change and motivation’ to try and encourage people into activity. Because everyone knows it’s good for them. But there are such significant barriers. We saw [the HPEP] as a signposting, to be honest, and then as we learnt more about it, we’re incorporating {the information] in our consultations with patients.’ [Interview, OCDS Clinical Lead]*

### Nurses’ beliefs about physical activity effectiveness

Beyond the factors of educational delivery, it is important to learn more about how receptive health professionals are to the concepts promoted within the HPEP. Figure 2 illustrates that individual factors e.g., knowledge, attitudes and beliefs, will influence how health professionals interpret the education delivered to them. For example, it is known that health professionals who exercise themselves, are more effective at communicating the exercise message because they translate their own beliefs, attitudes and behaviours to patients [25, 26]. Observational data of two HPEP presentations revealed a range of beliefs. The first presentation was delivered to District Nurses, where resistance to the patient exercise message was noted.

*The District Nurse training session became more interactive towards the end, where the ways in which nurses’ patients could be engaged in physical activity were discussed, including the use of the Generation Games DVD and the referral cards. The nurses engaged well. An interesting discussion covered the difficulties of patient behaviour change. There seemed to be some resistance to the idea that patients could indeed become more physically active due to some patients who continually defended their sedentary behaviour in relation to barriers* e.g., *‘I can’t do the suggested physical activity because of x’. Other nurses were given the opportunity to challenge this viewpoint and make suggestions for more productive approaches.* [*Observation Notes, District Nurse OCDS Training Course*]

During the second presentation, delivered to Practice Nurses, more positive comments were noted than during the first presentation, specifically in relation to practical tips and tools offered. The HPEP Clinical Lead believed that this difference in attitude was due to differing professional experiences of the two types of nurses, with District Nurses encountering more significant barriers to exercise because of the housebound nature of the patients they visited. The interactive discussion at the end of both HPEP sessions provided an opportunity to help health professionals re-evaluate these beliefs.

*‘Yes [the HPEP session] did [provide me with new information]. Having all the information that even the patients who are immobile can actually start.’ [Interview, Practice Nurse 3]*

**Fig. 4:**
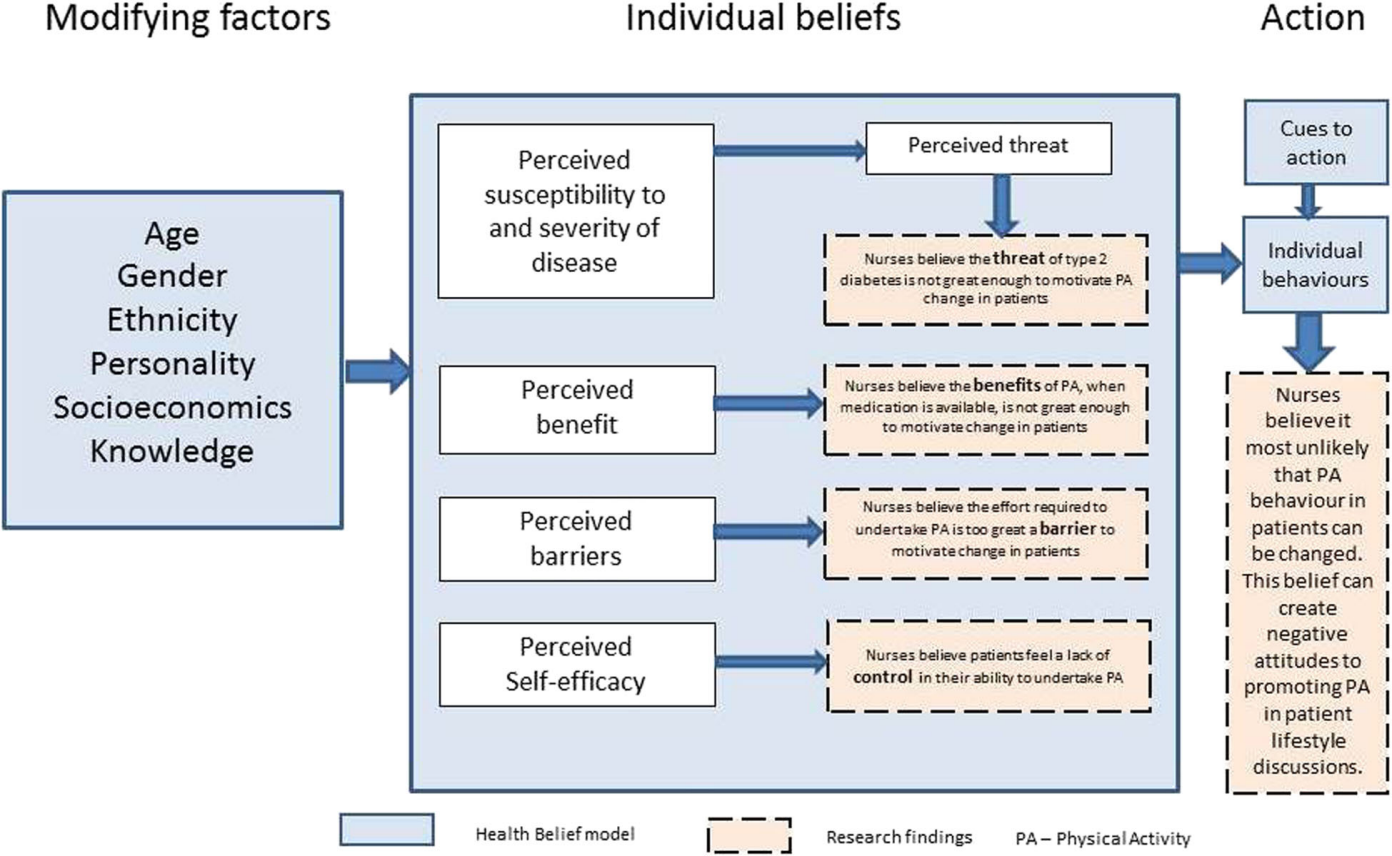
Health Belief model including findings showing nurses beliefs about patient attitudes to physical activity

Physical activity is recognised as one of the hardest health behaviours to change, perhaps because of the necessity for extensive planning and motivation in order to establish new habits [27]. It is helpful to try and understand beliefs within an established health behaviour model. The Health Belief model [28] (see Fig. 4) highlights four elements of relevance: *threat, benefit, barriers, self-efficacy*. The nurses interviewed in the case study, from four individual practices, were all responsible for discussing lifestyle changes with their diabetes patients following diagnosis by the GP (a system common amongst GP practices) [25]. All nurses interviewed held the belief that effecting patient physical activity change was very challenging. The model offers useful explanations for understanding beliefs about threat, benefit, barriers and self-efficacy and how they influence behaviour.

Type 2 Diabetes patients may not feel there is enough in it for them to motivate them to change behaviour, because the *‘perceived threat’* is not great enough.

*‘Because they’re comfortable… They’ve had it [type 2 Diabetes] a long time. They’re managing it to a degree.’ [Interview, Practice nurse 3]*

Type 2 diabetes patients will usually be prescribed medication to control the condition. Nurses believe that patients may see little *‘perceived benefit’* in the adoption of exercise, since medication will provide the same benefit.

*‘I think a large number of patients, there’s almost a sort of, well they’re on the pharmacology. They’ve got the statin… [diabetes] can be managed… It’s “Ah well, my blood sugar was a bit up, but I’m taking these tablets, so..”’ [Interview, Practice Nurse 4]*

All the nurses spoke of the effort required to increase physical activity as a *‘perceived barrier’*. Their own belief of the required effort was used to explain low patient take-up of exercise

*‘[They don’t do it] probably for the same reason I don’t do it. It’s too much effort. Hard work.’ [Interview, Practice nurse 2]*

*‘They [patients] know what they should be doing. It’s just ingrained lifestyle and it’s very hard to get people to change… there’s a perception that it’s easier to change the shopping basket and dietary habits than physically get off your bum and do stuff. Too much effort.’ [Interview, Practice nurse 1]*

One nurse highlighted her belief that for some patients, increased exercise was outside of their control – a lack of *‘perceived self-efficacy’* – that their situation was inevitable and personally unchangeable.

*‘Poor locus of control. They feel that they can’t change it [their physical activity level]. It’s inevitable…[Some patients think] “I’ve got to die from something.”’ [Interview, Practice nurse 4]*

### Changing the behaviour of health professionals

As stated above, the aim of the HPEP is firstly to educate about the benefits of exercise, in this case in relation to Diabetes, but then also to ask health professionals to put appropriate patients in touch with Generation Games for further physical activity advice. The emphasis at HPEP sessions is that very little effort is required on the part of the doctor or nurse

*‘It’s actually minimal, what [GPs] have got to do [to engage patients]. They’ve got to think about exercise, and introduce the concept. I try and explain to them that that doesn’t take more than a minute or two, because we will do the work for them. All they need to do is get the patient engaged enough to fill in a card [Generation Games sign-up card] and put it in the post or look at the website themselves.’ [Interview, HPEP Clinical Lead]*

However, after experience over two years of running the HPEP, it is clear to the Clinical Lead that in the large part, this hope has not been realised

*‘I started this project with a very idealistic view, ‘oh yes, we can just get out there and tell everyone and they’ll just do it’ [recommend patients to Generation Games]. But of course I’ve realised that some people do, but most [health professionals] don’t and it’s going to need much more investment to make a big difference.’ [Interview, HPEP Clinical Lead]*

**Fig. 5:**
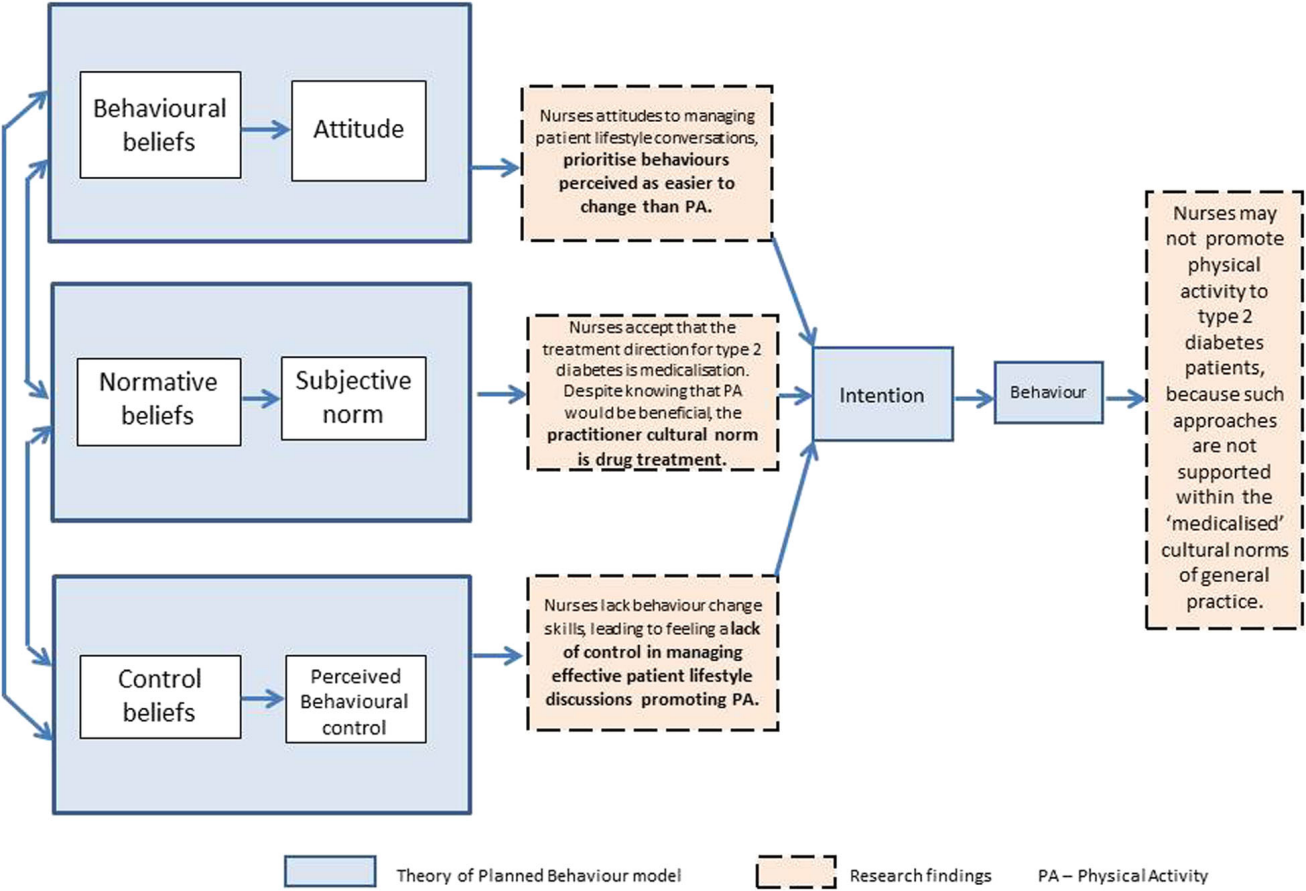
Theory of Planned Behaviour model including findings showing how cultural norms influence expectation and nursing practice

Despite the obvious benefits for patients, there are clearly barriers – beyond the individual beliefs mentioned above – which prevent health professionals offering ‘brief advice’ about physical activity, and signposting patients on to Generation Games. The Theory of Planned Behaviour [29] shown in Fig. 5 is a useful model for thinking about these reasons, suggesting three categories of belief (behavioural, normative and control) guiding any ‘intention to act’ – the precursor to the actual desired behaviour.

*‘Behavioural beliefs’* (personal beliefs and attitudes) in part guide the health professional as to whether or not they intend to a) discuss the benefits of exercise for their patients, and b) recommend them to contact Generation Games. As outlined in the earlier section above, all nurses stated their belief that physical activity uptake in their diabetic patients was unlikely for the majority. The beliefs about the challenge of increasing physical activity require nurses to adopt more ‘user-friendly’ terms

*‘…you say ‘we’ve got this fantastic exercise referral scheme’. They gloss over…I’ve stopped saying to people “exercise”. I say “are you quite active?” ‘[Interview, Practice nurse 1]*

This being the case, it is understandable that nurses should focus on what they believe patients perceive as more ‘achievable’ lifestyle change goals. It seems as though nurses ‘cherry pick’ what to tackle, focusing on the aspect which is likely to receive the most favourable response from patients

*‘No. You have a general discussion and then you get them to tell you what they think they could change…if they want to focus on one [behaviour] I would encourage them to maybe focus on [diet] first, and then come back to the others’ [Interview, Practice nurse 2]*

Within the context of the ‘lifestyle’ conversation which nurses have with diabetic patients therefore, it seems as though the intention to discuss exercise goals and then to go one step further to engage patients in considering Generation Games, is not prioritised because within the primary care setting, the pursuit of changing other behaviours is perceived as more realistic.

*‘Normative beliefs’* (their beliefs about whether ‘important others’ would approve or disapprove of the desired behaviour) can be understood as the influence of those related to their setting e.g., GPs, nurses, practice managers, patients, and other external guidance and influences. These ‘important others’ set the cultural environment within which the health professional works. It seems that practitioners believe that increased physical activity *would* be an effective treatment, albeit hard to achieve.

*‘In Diabetes2gether we say, in most cases most people with type 2 diabetes will end up on medication…but that lifestyle issues such as diet, weight management, activity, smoking cessation* etc. *should always be the fundamental building blocks.’ [Interview, OCDS Clinical Lead]*

The evidence here showed that the accepted treatment direction for those newly diagnosed shows a belief in medicalisation amongst practitioners. The procedure outlined in the following quote was common to all GP practices in which the nurses worked, and illustrates the procedure for use of medicines for diabetes control

*‘Then our protocol here is that we’ll give them 3 months to perhaps make some changes to diet, lifestyle, exercise…Get them in again after 3 months. Sit down, go through the results, see what changes they’ve been able to make, has that impacted on their actual blood tests. If we find the [blood result] has gone up or it’s too high, then we’ll instigate something like Metformin.’ [Interview, Practice nurse 1]*

Thus the ‘desired behaviour’ under such a protocol is not to prescribe physical activity; it is to prescribe medication, this being the prevailing ‘normative belief’ about controlling patient diabetes in reality.

*‘Control beliefs’* are factors that can facilitate or inhibit the desired behaviour in a practical way. Health professionals work within systems and routines which define how things are done in a given environment, and control beliefs held by any individual can govern how they perceive whether or not they are able to ‘make something happen’ in reality. There is a deficit of adequate training for health professionals in behaviour change techniques

*‘…there are some key skills round [motivational interviewing with patients] that I certainly haven’t had training on.’ [Interview, new OCDS Diabetes nurse]*

In addition to potentially feeling a lack of control because of inadequate training, nurses mentioned that they appreciated the HPEP session because it offered them tools to assist with feeling more in control of trying to encourage patients to consider increasing their exercise.

*‘You’ve got something for them physically to send off for [the DVD] and try it. As a nurse, it just makes you feel better that you are doing something. That you can give a solution that doesn’t involve too much effort. That’s great.’ [Interview, Practice nurse 1]*

### The exercise behaviour of people with type 2 diabetes

#### Life with a type 2 diabetes diagnosis

The confirmation of a type 2 diabetes diagnosis is by blood test, either routine (as part of a screening programme) or requested by the patient. It is quite common in the early stages of type 2 diabetes for there to be no symptoms [30]. One striking feature of the patients’ interviews, is how low down on their list of priorities they place their diabetes within the context of their physical capabilities. All patient interviewees mentioned limitations on what they were able to do, but because of other conditions, not diabetes.

*‘I don’t suffer effects of diabetes.… Having had both knees replaced, I can’t run, I can’t walk quickly, I can’t kneel.’ [Interview, Patient 1]*

*‘I had a stroke 3 months ago…This time I lost my left side…they found I had an irregular heartbeat. It’s been a traumatic 6 months, so I haven’t done much diabetes-wise.’ [Interview, Patient 3]*

*‘Well at the moment it’s my heart which is stopping me [from being more physically active]. I am more bothered about my heart condition than the diabetes. It’s my breathing which is stopping me…The diabetes itself, I have controlled with changes to diet, so in a way it’s not that which is a difficulty to me.’ [Interview, Patient 4]*

One difficulty may be related to the nature of type 2 diabetes, which in the early stages, may mean patients do not feel unwell. Added to this, is the knowledge that medication is available, and can be perceived to some extent as the ‘solution’ – a view which may work against the effectiveness of health promotion messages. Within the context of Self Determination Theory [31], this situation has been entitled ‘amotivation’ – a condition where ‘the person sees no connection between the action and the desired outcomes’ (p114), as suggested in the following quote.

*‘There’s this temptation to think “I feel fine”. So then why should I go to a whole load of trouble [making lifestyle changes] to prevent complications when they may not happen.’ [Interview, OCDS Clinical Lead]*

**Fig. 6:**
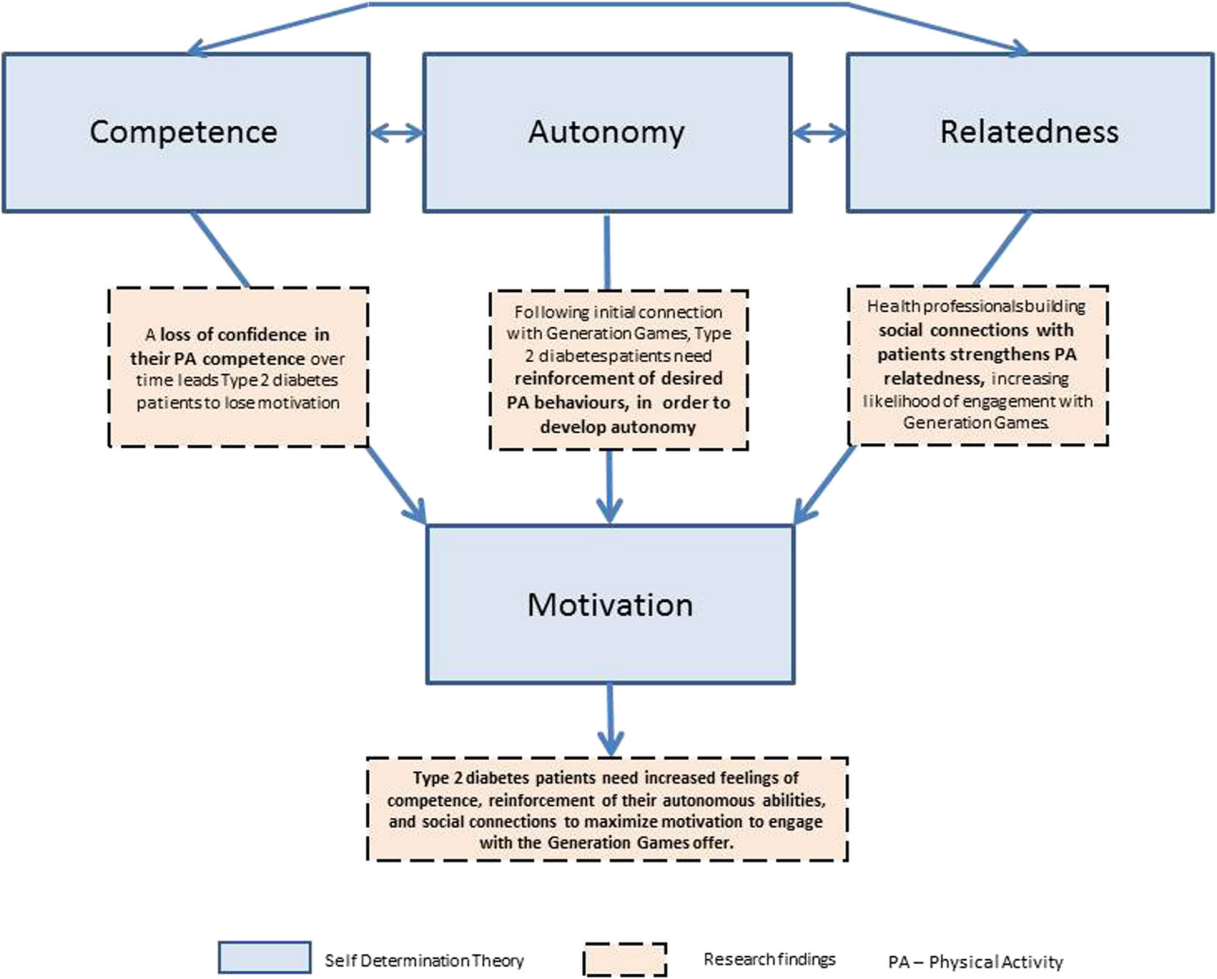
Self Determination Theory model including findings relating to competence, relatedness and autonomy which affect motivation

Self Determination Theory (see Fig. 6) is a helpful model for understanding why the patients in this case study – who know they should exercise more – do not. It posits that there are three basic needs which support motivation to act – Competence, Relatedness and Autonomy.

### Competence

Patient physical activity levels showed a clear decline over time – three out of the four participants described this, and the physical reasons to explain the behaviour. All participants, prior to signing up to Generation Games, were aware that they needed to be more physically active. In the context of Competence therefore, patient interviewees spoke of their current physical activity in relation to the loss of abilities over time, using phrases like ‘I used to be able…’, ‘since my operation I haven’t been able to…’, ‘can’t do as much as we once did…’ There was a sense of losing confidence because they were experiencing a loss of effectiveness, bringing an overall feeling of a lack of competence.

*‘[I used to be] quite active. I played some tennis… I did keep fit classes once a week and swimming…But having had trouble with my ankle…I don’t do as much walking as I once did…I miss that more than the keep fit because the keep fit I had to stop because of the ankle problem. I do get pain in my feet…and then I do think, ‘Oh, a walk to the shops, OK.’ But I don’t want to go the extra bit further…I’m perhaps a bit lazy about finding somewhere to go to a Keep Fit [class].’ [Interview, Patient 2]*

*‘[My current level of physical activity is] zero…My biggest problem is now [after getting home from work]. Coz I sit here [on the sofa] now till I go to bed…I can’t be bothered. There’s nothing for me to do…My husband [says to me] sit down, sit down, you look tired. So that’s what I do.’ [Interview, Patient 3]*

### Autonomy

This domain refers to self-regulation, to the sense of fully assenting. In terms of what these patient participants expected Generation Games to deliver, none appeared autonomous. The engagement with Generation Games was because, in all cases, a health professional had advised them of its value – either during a one-to-one session with a doctor or diabetes nurse, or during a 3-hour structured education session – diabetes2gether – a programme taken up by 50% of Oxfordshire’s newly diagnosed diabetes patients and attended by all four patient interviewees. There was a sense of being ready to be ‘told what to do’.

*‘At the course it [Generation Games] was just mentioned, and if you’d like a DVD, fill in this card, I think, and they will give you exercises to do.’ [Interview, Patient 2]*

*‘She [the diabetes2gether nurse] told me to [request the Generation Games DVD]. She said it was quite interesting…I remember them saying it was a good thing to have.’ [Interview, Patient 3]*

This evidence is strengthened by the patient comments when asked of their experience of the Generation Games DVD, which demonstrate that in terms of effecting physical activity behaviour change, older people may need effective communication and re-inforcement of desired behaviours in addition to receiving the tools to assist them, such as the DVD.

*‘I’ve got it in the house somewhere, but I haven’t played it. I must have ticked the box requesting it.’ [Interview, Patient 4]*

### Relatedness

This aspect of Self Determination Theory suggests that ‘relatedness’ – or the extent to which an individual receives support, and feels connected and included by others - facilitates being intrinsically motivated. Within the domain of exercise, activities will be pursued and enjoyed in part as a result of values and skills conveyed by significant others e.g., a diabetes nurse, doctor or other exercise professional. When those needs are not met, motivation will be lost and individuals will be less likely to maintain exercise behaviours [31].

For all four patient interviewees, there was little evidence of that kind of support for any exercise regimes. Two interviewees mentioned health professionals in places where they had previously lived, who had taken a personal interest, built up a relationship of trust, which had resulted in both making the lifestyle changes recommended.

*‘There was a diabetic nurse there who was excellent, and she got me started on the right way to live. She used to say, “Mrs X, you’ve got to cut down on sugar…no, no, no sugar.” She was quite fierce, but she was lovely…I lost weight with the dieting, and the extra walking as well [which helped me to control the diabetes].’ [Interview, Patient 2]*

Since the removal of these support mechanisms, both interviewees reported a decrease in physical activity. One further interviewee was undergoing a cardiac rehabilitation programme, successfully increasing her physical activity under the guidance and support of the staff. However, the fourth interviewee had not experienced any support for exercise. She described the complexity of trying to alter her poor health behaviours, where the diabetes nurse discussed with her the priority for smoking cessation over and above attention to diet or exercise needs.

In considering whether or not Generation Games can fulfil the ‘relatedness’ support role, it is interesting to note that despite all four patient interviewees having been interested enough to complete the Generation Games card, neither the arrival of the DVD nor the list of exercise opportunities locally was sufficient in themselves to engage interest and lead to a change in exercise behaviour. Given the array of known factors affecting how those with chronic conditions struggle to maintain physical activity, even once it has been adopted, this is not a surprising finding. Health professionals need training to facilitate an exercise maintenance plan for each patient, addressing individually presented barriers in order to support and encourage effectively [32].

Self Determination Theory illuminates the experience of these patient interviewees, to show that their belief in their own competence and their own self-regulation of activity require greater support and encouragement in order to facilitate any positive change in exercise behaviour. Such aspects may be beyond the scope of Generation Games per se, but not necessarily beyond the scope of health professionals who work with diabetes patients day to day, and who have reacted positively to the training and tools offered by the Generation Games service to assist them in the challenging task of increasing patient physical activity.

## Discussion

The study research question and findings set out under three themes are summarised below:

### Research Question

‘How can we understand the processes at work, from health professional engagement with the HPEP to increasing the physical activity of Generation Games participants.’

### Finding 1 Health professional engagement with the HPEP

GPs appear more resistant to engagement with the HPEP than those healthcare professionals directly promoting physical activity to patients. Effective programme delivery requires enthusiastic tutors who communicate the exercise risk reduction message with an interactive style, particularly given such resistance. In addition, health professionals believe that efforts to change the exercise behaviour of their type 2 diabetes patients will be ineffective due to negative perceptions held by those patients about success likelihood. Such attitudes pose a challenge to communicating the physical activity message central to the HPEP.

### Finding 2 Changing the behaviour of health professionals

The cultural environment of general practice provides little support to the promotion of exercise for the type 2 diabetes patient. During patient lifestyle discussions, nurses’ beliefs about exercise lead them to prioritise other behaviours, e.g., diet, where behaviour change is perceived as more likely. Primary care settings normalise the ‘medicalisation’ of type 2 diabetes treatment, which may shift priorities in relation to promoting exercise behaviour change. Thus we have found that the norms of primary care settings fail to adequately support efforts to promote physical activity as either prevention or therapy.

### Finding 3 The exercise behaviour of people with type 2 diabetes

Since type 2 diabetes is progressive, those in the early stages with few symptoms may not see the connection between exercise and improving their condition. Promotion of physical activity for such patients may be further hindered, since people with type 2 diabetes may lose competence in their own exercise ability, perhaps creating expectations that Generation Games might ‘tell them what to do’. We have shown that type 2 diabetes have increased their exercise in the past, when health professionals have provided considerable on-going support.

### A model of good practice

**Fig. 7:**
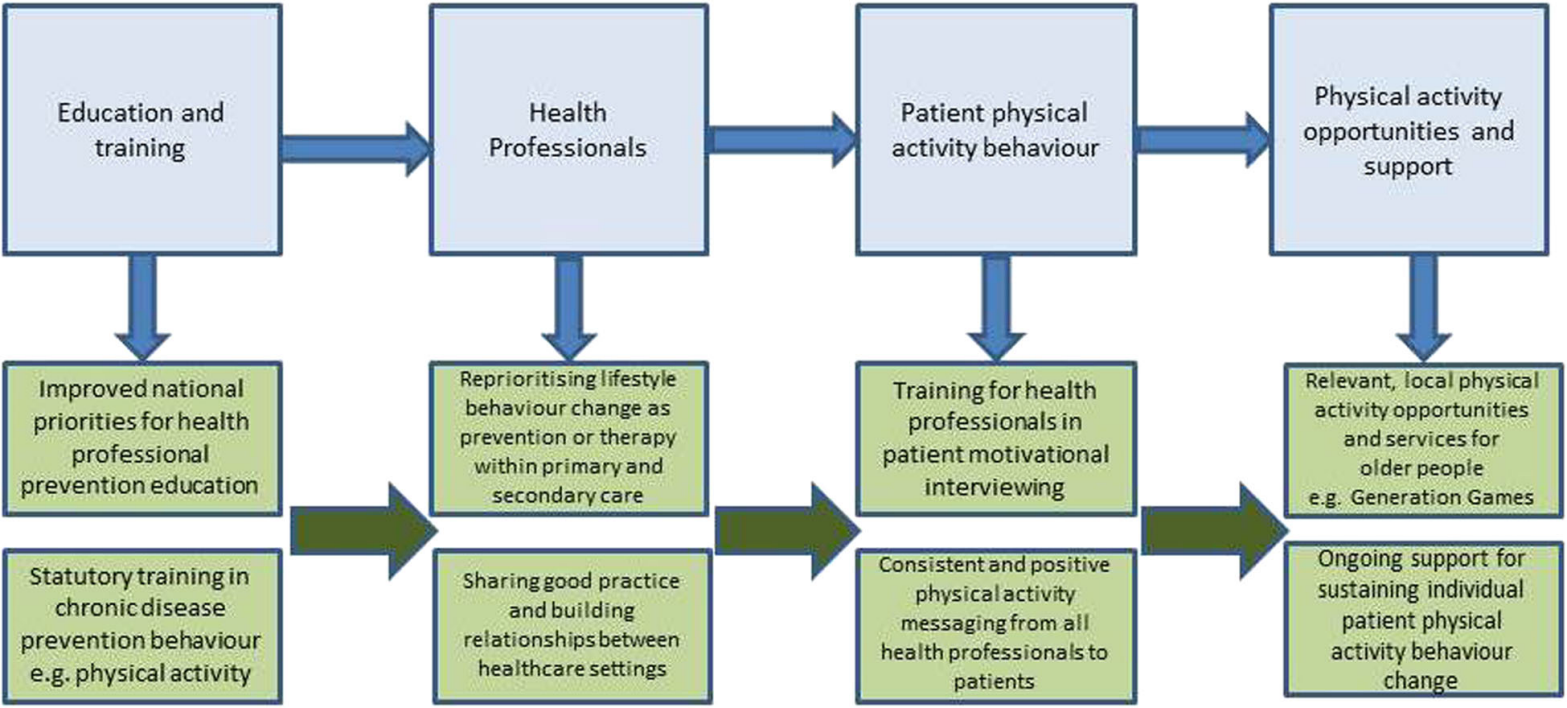
Model of good practice for improving physical activity promotion to chronically ill older patients within primary and secondary healthcare settings

Patient engagement with exercise for a wide range of diseases including type 2 diabetes is highly desirable, given the strong evidence of its health benefits. Continuing professional development promoting exercise medicine for health professionals is not routine within the NHS. Suggestions have been made in this case study that referral or recommendation of exercise to patients by primary care health professionals receives a low priority, for a range of reasons outlined here. By instigating the HPEP, Generation Games has begun to raise the profile of exercise as therapy or prevention within Oxfordshire NHS. One outcome has been the demonstration that successful relationships can be developed as secondary care bodies like the Oxfordshire Community Diabetes Service recognise the value of the HPEP offer, build relationships, and share good practice by promoting key health messages and sustainable ways for patients to achieve behaviour change by engaging with realistic physical activity opportunities such as those provided by Generation Games. This case study has shown that such links are achievable and form part of a good practice model for physical activity promotion which we now suggest (see Fig. 7), and which could be adopted by others.

### Changing primary care exercise culture

Referring back to Fig. 3, it can be noted that the HPEP requires a number of changes to health professional behaviour prior to changing patient exercise behaviour. The second section of the Findings described the challenge of changing health professional culture. Practice nurses, faced with the newly diagnosed type 2 diabetes patient, describe how physical activity is the hardest patient behaviour to change. Such patients may present with multiple unhealthy behaviours, and it is no surprise that behaviours such as diet or smoking may be chosen above exercise as those to be addressed, since the achievable change likelihood is greater. Nurses spoke of the support available within practice life for tackling such behaviours i.e., smoking cessation medication and services, dietician services.

Thus, the OCDS values the ‘tools’ which the HPEP promotes to nurses such as the DVD and the exercise signposting service offered to Oxfordshire patients, because the primary care setting within which they work currently provides little to support exercise behaviour change. These ‘tools’ offer such nurses a ‘way in’ for them as part of any exercise discussion with patients. When such tools exist, the cultural acceptance of devoting valuable time talking to patients about their physical activity becomes more valid since credible local opportunities can be presented. Figure 7 illustrates how a more integrated approach, including reprioritising the value of physical activity as prevention or therapy within healthcare settings and providing training to support it, would create a more helpful environment within which such work could thrive.

### Effective exercise support for those with a chronic illness

It could be argued that the fact that no discernible increase in exercise was reported by the interviewed patients in this case study, suggests that the ‘reach’ of the HPEP to the patient was ineffective. This case study highlighted the complexity of that ‘reach’ (see Fig. 1). The study described patient interviewees’ physical activity decline: a phenomenon commonly prevalent in older adults generally. It described the progressive nature of type 2 diabetes which, for those with multiple conditions, may persuade some patients – as alluded to under the Health Belief Model – that exercise behaviour change offers too few benefits in relation to their type 2 diabetes. It also describes how when health professional support has been in place in the past, patient interviewee exercise behaviour was changed and increased.

Thus, the argument here is that such support is an essential pre-requisite to successful exercise behaviour change. GG was successful at ‘delivering the information’. However, research indicates that information alone may not change behaviour, particularly for those with specific needs. Expert recommendations indicate that specific intervention strategies to help sub-groups such as those with chronic illness, are necessary in order to maintain attempts to be physically active, since such attempts are often derailed as a result of coping with chronic illness or injury [33]. Successful interventions need to contain a combination of behavioural and/or cognitive tools i.e., goal-setting self-monitoring, feedback, support, relapse-prevention training [34], as suggested by the good practice model (see Fig. 7).

### Limitations

The health professionals interviewed in the study were largely nurses – either connected directly to the OCDS or practice nurses attending a HPEP session. The study would have been strengthened by having at least one GP who could have described from experience working in their setting, and could have offered their views of engaging patients in exercise behaviour change, and their outlook on the HPEP and GG offer in general. Budgetary restrictions limited the study to a small scale, and therefore limited the numbers of health professionals and patients recruited. A larger number of both would have enabled a broader range of perspectives to be gathered. The use of theoretical models within the case study was a useful means of framing and interpreting data, but the use of different models or none may have yielded alternative interpretations. Reflexivity is an important aspect of qualitative research, described as a process of ‘self-examination’ in order to be aware of and address researcher subjectivities [35]. In this study, there was only one researcher who collected all the data, including observations and interviews. Despite all observation notes being discussed with the HPEP tutors, and interviews subsequently checked with interviewees, there were no additional researchers who could have discussed notes and transcripts. However, the process of sharing and modifying findings with fellow authors who are practitioners in the field did assist reflexivity. Nevertheless we recognise these potential researcher biases as further limitations.

## Conclusion

The findings indicate that firstly it is possible to provide health professional education, promoting the adoption of physical activity, which is so well regarded that it is adopted by the health professional community into its own practice. Such has been the case for Generation Games’ HPEP in respect of the Community Diabetes Service within Oxfordshire NHS. Secondly, GG have succeeded in offering local, practical tools to support health professionals in their efforts to encourage type 2 diabetes patients to exercise more. Thirdly, GG have demonstrated that, with a supportive partner such as the OCDS, contact can be made with interested patients. The lack of any discernible physical activity increase for the patient participants in this case study serves to illustrate the complex nature of changing behaviour.

Two implications follow as part of any future action plan. The first would be to recommend the establishment of robust partnerships and processes, which can deliver comprehensive education and training to health professionals. The HPEP reaches those who express an interest within Oxfordshire, but for a broader public health effect nationally, provision should incorporate an adequately funded physical activity promotion education and training delivery mechanism within the NHS available to all relevant practitioners. The second would be that, although education has the potential to change health professional behaviour in the ways indicated above, changing patient exercise behaviour requires a more coordinated and systematic approach which places the patient at the centre and provides a platform for on-going support. Organisations such as Generation Games working alongside others who have this patient-centred capacity, such as health professionals, might then be able to deliver a more targeted, supportive and therefore more effective approach to increasing physical activity in older people with chronic long-term illness.

## Abbreviations

GG: Generation Games
HPEP: Health Professional Education Programme
NHS: National Health Service
NICE: National Institute for Health and Clinical Excellence
OCDS: Oxfordshire Community Diabetes Service

## References

[1] Lee I-M, Shiroma EJ, Lobelo F, Puska P, Blair SN, Katzmarzyk PT (2012). Effect of physical inactivity on major non-communicable diseases worldwide: an analysis of burden of disease and life expectancy. Lancet.

[2] Joint Health Surveys Unit (2013). Health Survey for England 2012: Health, social care and lifestyles.

[3] Royal College of Physicians. Falling Standards, broken promises: report of the national audit of falls and bone health in older people 2010. 2011. https://www.rcplondon.ac.uk/file/4356/download?token=GEWifpJb. Accessed 23 Jan 2017.

[4] Hauner H, Holt RIG, Cockram CS, Flyvbjerg A (2010). Obesity and Diabetes. Textbook of Diabetes.

[5] Diabetes UK. Position statement: Prevention of Type 2 diabetes: reducing risk factors. 2012. https://www.diabetes.org.uk/Documents/Position%20statements/diabetes-uk-position-statement-preventing-type-2-0513.pdf. Accessed 27 June 2016.

[6] Hamman RF, Wing RR (2006). Effect of weight loss with lifestyle intervention on the risk of diabetes. Diabetes Care.

[7] National Institute for Health and Clinical Excellence. Physical Activity: brief advice for adults in primary care. NICE Public Health Guidance 44. 2013

[8] Mooney H (2012). Doctors are told to “make every contact count” to reduce costs of poor lifestyles. BMJ.

[9] Nunan D (2016). Doctors should be able to prescribe exercise like a drug. BMJ.

[10] Hébert ET, Caughy MO, Shuval K (2012). Primary care providers' perceptions of physical activity counselling in a clinical setting: a systematic review. Br J Sports Med.

[11] Weiler R, Jones N, Hutchings K, Stride M, Adejuwon A, Baker P, Larkin J, Chew S. Sports and Exercise Medicine: A Fresh Approach. NHS Sport and Exercise Medicine Services. 2011

[12] Killoran A, Jagroo J, Chatterton H, Ellis S (2013) NICE Public Health Guidance Update. Journal of Public Health DOI: http://dx.doi.org/10.1093/pubmed/fdt090 10.1093/pubmed/fdt090 23978845

[13] Weiler R, Jones N, Hutchings K, Stride M, Adejuwon A, Baker P, Larkin J, Chew S. Sports and Exercise Medicine: A Fresh Approach. NHS Sport and Exercise Medicine Services; 2011

[14] Lawrence W, Black C, Tinati T, Cradock S, Rufia B, Jarman M, Pease A, Margetts B, Davies J, Inskip H, Cooper C, Baird J, Barker M (2016). ‘Making every contact count’: evaluation of the impact of an intervention to train health and social care practitioners in skills to support health behaviour change. J Health Psychol.

[15] Davis DA, Thomson MA, Oxman AD, Haynes RB (1995). Changing physician performance: a systematic review of the effect of continuing medical education strategies. JAMA.

[16] Swann C, Carmona C, Ryan M, Raynor M, Barış E, Dunsdon S, Huntley J, Kellet MP. Health systems and health- related behaviour change: a review of primary and secondary evidence, National Institute for Health and Clinical Excellence World Health Organization Europe. 2010

[17] Yin R (1994). Case study research: Design and methods.

[18] Moore G, Audrey S, Barker M, Bond L, Bonell C, Cooper C, Hardeman W, Moore L, O’Cathain A, Tinati T, Wight D, Baird J (2014). Process evaluation in complex public health intervention studies: the need for guidance. J Epidemiol Community Health.

[19] Patton MQ (2002). Qualitative research and evaluation methods.

[20] Creswell JW (2007). Qualitative Enquiry and Research Design: Choosing Among Five Approaches.

[21] Maxwell JA (1992). Understanding and validity in qualitative research. Harv Educ Rev.

[22] Meyer CB (2001). A case in case study methodology. Field Methods.

[23] Weiler R, Stamatakis E (2010). Physical activity in the UK: a unique crossroad?. Br J Sports Med.

[24] National Institute for Health and Clinical Excellence. Physical Activity: brief advice for adults in primary care. NICE Public Health Guidance 44; 2013

[25] Mckenna J, Naylor PJ, McDowell N (1998). Barriers to physical activity promotion by general practitioners and practice nurses. Br J Sports Med.

[26] Frank E, Kunovich-Frieze T. Physicians’ prevention counseling behaviours; current status and future directions. Prev Med. 1995;24:543–5. 10.1006/pmed.1995.1086 8610075

[27] Dishman RK, Heath GW, Lee IM (2003). Physical Activity Epidemiology.

[28] Glanz K, Rimer BK, Viswanath K (2008). Health Behavior and Health Education. Theory, Research and Practice.

[29] Ajzen I (1991). The theory of planned behaviour. Organ Behav Hum Decis Process.

[30] National Institute of Diabetes and Digestive and Kidney Diseases. http://diabetes.niddk.nih.gov/dm/pubs/diagnosis. Accessed 27 June 2016.

[31] Ryan RM, Williams GC, Patrick H, Deci EL (2009). Self-determination theory and physical activity: The dynamics of motivation in development and wellness. Hell J Psychol.

[32] Scott S, Breckon J, Copeland R, Hutchison A (2015). Determinants and strategies for physical activity maintenance in chronic health conditions: a qualitative study. J Phys Act Health.

[33] King AC, Rejeski WJ, Buchner DM (1998). Physical activity interventions targeting older adults. A critical review and recommendations. Am J Prev Med.

[34] Chase JD (2013). Physical activity interventions among older adults: a literature review. Res Theory Nurs Pract.

[35] Ahern K (1999). Ten tips for reflexive bracketing. Qual Health Res.

